# Emergency Resection for Colonic Cancer Has an Independent and Unfavorable Effect on Long-Term Oncologic Outcome

**DOI:** 10.1007/s12029-024-01074-y

**Published:** 2024-07-31

**Authors:** Marta Sandini, Stefania Piccioni, Simona Badalucco, Eleonora Andreucci, Margherita Gambelli, Andrea Fontani, Riccardo Piagnerelli, Luigi Verre, Daniele Marrelli, Franco Roviello

**Affiliations:** Department of Medicine, Surgery and Neurosciences, Unit of General Surgery and Surgical Oncology, University of Siena, Strada delle Scotte, 4, Siena, 53100 Italy

## Abstract

**Background:**

Long-term outcomes in patients undergoing emergency versus elective resection for colorectal cancer (CRC) remain controversial. This study aims to assess short- and long-term outcomes of emergency versus elective CRC surgery.

**Methods:**

In this single-center retrospective cohort study, patients undergoing emergency or elective colonic resections for CRC from January 2013 to December 2017 were included. Primary outcome was long-term survival. As secondary outcomes, we sought to analyze potential differences on postoperative morbidity and concerning the oncological standard of surgical resection. The Kaplan-Meier curves and Cox proportional hazard model were used to compare survival between the groups.

**Results:**

Overall, 225 CRC patients were included. Of these 192 (85.3%) had an elective and 33 (14.7%) an emergency operation. Emergency indications were due to obstruction, perforation, or bleeding. Patients in the emergency group had higher ASA score (*p* = 0.023), higher Charlsson comorbidity index (CCI, *p* = 0.012), and were older than those in the elective group, with median age 70 (IQR 63–79) years and 78 (IQR 68–83) years, for elective and emergency, respectively (*p* = 0.020). No other preoperative differences were observed. Patients in the emergency group experienced significantly more major complications (12.1% vs. 3.6%, *p* = 0.037), more anastomotic leakage (12.1% vs. 1.6%, *p* = 0.001), need for reoperation (12.1% vs. 3.1%, *p* = 0.021), and postoperative mortality (2 patients vs. 0, *p* < 0.001). No differences in terms of final pathological stage, nor in accuracy of lymphadenectomy were observed. Overall survival was significantly worse in case of emergency operation, with estimated median 41 months vs. not reached in elective cases (*p* < 0.001). At the multivariate analysis, emergency operation was confirmed as independent unfavorable determinant of survival (with hazard rate HR = 1.97, *p* = 0.028), together with age (HR = 1.05, *p* < 0.001), postoperative major morbidity (HR = 3.18, *p* = 0.012), advanced stage (HR = 5.85, *p* < 0.001), and need for transfusion (HR = 2.10, *p* = 0.049).

**Conclusion:**

Postoperative morbidity and mortality were increased in emergency versus elective CRC resections. Despite no significant differences in terms of accuracy of resection and pathological stages, overall survival was significantly worse in patients who underwent emergency procedure, and independent of other determinants of survival.

## Introduction

Colorectal cancer (CRC) is the commonest malignancy in Western countries, and the second leading cause of cancer-related mortality [1]. Despite screening programs for early detection of CRC, the rate of patients with an emergency presentation remains not negligible, about one-fourth of all cases [2, 3]. The most frequently reported symptoms related to the emergency presentation are bowel occlusion, bleeding, and perforation [4, 5].

An association between the emergency presentation and a higher likelihood of colonic location of the neoplasm—compared to the rectal site—has been broadly described [6]. Furthermore, the emergency presentation of CRC may affect short- and long-term outcomes, which appears to be worse in comparison to elective cases [7, 8]. Advocated factors justifying these findings include more advanced disease stage, more lymphovascular and vascular invasion, poorly differentiated tumors, higher American Society of Anesthesiology scores at presentation, and increased systemic inflammatory response in emergency cohorts [6, 9]. However, data are far to be univocal according to the independent effect of the emergent operation on long-term oncologic outcomes [10]. Also, a case-matched analysis adjusting for confounders such as age, gender, stage, and receipt of adjuvant therapy failed to prove a dismal effect of urgent surgery on overall survival [11].

The aim of this study was to assess whether patients who underwent emergency surgery for CRC had a more unfavorable long-term prognosis compared to a contemporary cohort of elective patients. As secondary outcome, we analyzed potential differences in terms of technical adequacy of the surgical resection according to international standard in both elective and emergency procedures.

Given the differences between colon and rectal adenocarcinomas in terms of molecular carcinogenesis, pathology, surgical topography, multimodal treatment, and long-term outcomes [12, 13], only colon cancer patients were included in the analysis.

## Methods

All consecutive patients who underwent colonic resections at our Institution, between January 2013 and December 2017 were retrospectively included. Exclusion criteria were palliative interventions without a curative intent. We also not considered in our analysis those CRC tumors with extraperitoneal extent, for the reasons stated above.

Patient demographic and clinical data were extracted from the institutional database and from electronic patient charts. Given the retrospective nature of the present study, no approval of the local ethical committee is required, according to the Italian legislation. The following information were collected for both the elective and emergency cohorts: age, gender, body mass index (BMI), American Society of Anesthesiology (ASA) score, Charlson Comorbidity Index (CCI), the operative procedure and technique (open or minimally invasive), the strategy with primary anastomosis, or protective, divertive, or definitive stoma, the site of the neoplasm, the final pathology, and the duration of hospitalization. The onset of any postoperative complications was also collected, and graded per the Clavien Dindo Classification (CDC) and the Comprehensive Complication Index (CCI) scales [14, 15]. Follow-up was made through outpatient visit and phone interviews. If death was not reported during the follow-up period, patients were censored at the last available contact date.

### Statistical Analysis

Normal distribution of continuous variables was evaluated at the Kolmogorov-Smirnov test. Data are expressed as median and interquartile range (IQR). The Mann-Whitney *U*-test was used for continuous variables. Nonrandom association for categorical variables was tested with the Fisher’s exact test.

### Survival Analysis

The Kaplan-Meier log-rank (Mantel-Cox) and the univariate Cox-proportional hazard method were used to analyze potential differences in overall survival according to type of surgery either elective, or emergency. A subgroup analysis was run according to the onset of severe postoperative complications—namely with CDC score of 3 or more—or not.

A Cox proportional hazard model was built to assess factors independently associated with OS. The following variables were included in the model: age, the Charlson Comorbidity Index (CCI), the onset of severe complication, the stage at final pathology, the receipt of perioperative transfusions and the presence of definitive stoma. All those variables are well-known recognized predictors of survival following resection for colonic cancer and may represent confounders according to the elective or emergency timing of resection. The realization of permanent stoma may express either technical difficulties during surgery, or a more compromised clinical status of the patient.

For each test, a two-sided *p*-value of 0.05 was considered significant. All computations were made with the IBM Corp. Released 2021. IBM SPSS Statistics, Version 28.0. Armonk, NY, USA.

This study was conducted according to the Strengthening the Reporting of Observational Studies in Epidemiology (STROBE) guidelines for observational studies available at http://www.strobe-statement.org.

## Results

Overall 225 patients over a 5-year period were included in the analysis. The patients were divided into elective (192, 85.3%) and emergency (33, 14.7%). Indications for emergency colectomy were occlusion in 22 (66.7%), 5 (15.2%) perforations, and 4 (12.1%) intraluminal bleeding. The pre- and intraoperative characteristics of the patients are depicted in Table 1. Emergency patients were generally older (median age 78 vs. 70 for emergency and elective, respectively, *p* = 0.020), had higher prevalence of ASAIII-IV (*p* = 0.023), and higher Charlson Comorbidity Index scores (CCI, *p* = 0.012). All patients in the emergency group underwent open surgery, while 37% of the elective group had minimally invasive operations, we also observed significantly more Hartmann procedures in the emergency group.

**Table 1:** Pre- and intraoperative characteristics of the study population

	**Elective** ***N*** **= 192**		**Emergency** ***N*** **= 33**		***p*-value**
	**N/median**	**%/IQR**	**N/median**	**%/IQR**	
**Age**	70	63–79	78	68–83	**0.020**
** ASA III-IV**	50	26%	15	45.5%	**0.023**
** Smoking habits**	27	14.1%	4	12.1%	0.757
**Chronic cardiac disease**	43	22.4%	12	36.4%	0.085
** Diabetes**	31	16.1%	6	18.2%	0.771
** COPD**	17	8.9%	4	12.1%	0.551
**Charlson Comorbidity Index**					**0.012**
0–2	7	3.7%	3	9.1%	
3–4	48	25%	2	6.1%	
5–7	102	53.1%	16	48.5%	
≥8	35	18.2%	12	36.4%	
**Preoperative Hb (g/dL)**					0.214
** >12**	115	59.9%	25	75.8%	
** 12−8**	70	36.5%	7	21.2%	
** <8**	7	3.6%	1	3.0%	
**Symptoms at presentation**					
Occlusion			22	66.7%	
Perforation			5	15.2%	
Bleeding			4	12.1%	
**Site of the neoplasm**					0.364
Right colon	101	52.6%	13	39.4%	
Transversum	12	6.3%	3	9.1%	
Left colon	79	41.1%	17	51.5%	
**Surgical technique**					**<0.001**
Open	121	63.0%	33	100%	
Minimally invasive	71	37.0%	0	0%	
**Surgical PROCEDURE**					**<0.001**
Right hemicolectomy	105	54.7%	15	45.5%	
Transverse resection	11	5.7%	1	3%	
Left hemicolectomy	34	17.7%	3	9.1%	
Sigmoidectomy	40	20.8%	6	18.2%	
Hartmann	2	1,1%	8	24.2%	
**Anastomosis**					0.054
Manual	106	55.8%	19	76%	
Mechanical	84	44.2%	6	24%	
**Permanent stoma**					**<0.001**
0	188	97.9%	25	75.8%	
1	4	2.1%	8	24.2%	
**Loop ileostomy**					0.289
0	165	85.9%	26	78.8%	
1	27	14.1%	7	21.2%	
**Surgical Time (min)**	190	150–240	180	150–210	0.308

Overall morbidity, medical and surgical morbidity were not dissimilar in the two groups (Table 2).

**Table 2:** Postoperative complications in the study population

	**Elective** ***N*** **= 192**		**Emergency** ***N*** **= 33**		***p*-value**
	**N/median**	**%/IQR**	**N/median**	**%/IQR**	
**Total complications**					0.247
0	141	73.4%	21	63.6%	
1	51	26.6%	12	36.4%	
**Medical complications**					0.830
0	160	83.3%	27	81.8%	
1	32	16.7%	6	18.2%	
**Surgical complications**					0.171
0	171	89.1%	26	78.8%	
1	21	10.9%	7	21.2%	
**Major complications (Clavien Dindo ≥ 3)**					**0.037**
0	185	96.4%	29	87.9%	
1	7	3.6%	4	12.1%	
**Anastomotic leakage**					**0.001**
0	189	98.4%	29	87.9%	
1	3	1.6%	4	12.1%	
**Comprehensive Complication Index**	0	0-8.7	0	0-20.9	0.085
**Reintervention**					**0.021**
0	186	96.9%	29	87.9%	
1	6	3.1%	4	12.1%	
**Postoperative trasfusions**					**0.022**
0	183	95.3%	28	84.8%	
1	9	4.7%	5	15.2%	
**Average length of stay (days)**	8	7–10	12	9–15	**<0.001**
**Perioperative mortality**					**<0.001**
0	192	100%	31	93.9%	
1	0	0%	2	6.1%	

Despite similar median CCI, the occurrence of major complications was significantly higher in the emergency group (12.1% vs. 3.6%, respectively; *p* = 0.037), as well as postoperative mortality (0% vs. 6.1%, *p* < 0.001). Similarly, the occurrence of anastomotic leakage, the need for postoperative transfusions and reintervention was significantly increased in the emergency group (*p* = 0.001, *p* = 0.021 and 0.022, respectively). Consequently, also the duration of hospitalization was median 4 days longer in the emergency group (*p* < 0.001). No differences were detected in the receipt of adjuvant chemotherapy.

At the final pathology, no differences in terms of stage, grading, type, and resection margins were detected, as per Table 3. Notably, no differences according to the number of lymph-nodes resected were detected (median 19 vs. 18, in elective versus emergency respectively), and met the oncological standards in overall 87.4% of cases.

**Table 3:** Final pathology and postoperative treatments

	**Elective** ***N*** **= 192 (85.3%)**		**Emergency** ***N*** **= 33 (14.7%)**		***p*-value**
	**N/median**	**%/IQR**	**N/median**	**%/IQR**	
**Grading**					0.344
G1	1	0.5%	1	3.0%	
G2	101	52.6%	18	54.5%	
G3	90	46.9%	14	42.4%	
**Histotype**					0.565
Mucinous	66	34.9%	10	30.3%	
Adenocarcinoma	125	65.1%	23	69.7%	
**Resection margins**					0.701
R0	187	97.4%	32	97%	
R1	2	1.0%	0	0%	
R2	3	1.6%	1	3.0%	
**Stage**					0.429
0	2	1.0%	0	0%	
I	41	21.4%	1	3.0%	
II	67	34.9%	12	36.4%	
III	47	24.5%	12	36.4%	
IV	35	18.2%	8	24.2%	
**T**					0.142
is	2	1.0%	0	0%	
1	12	6.3%	0	0%	
2	37	19.3%	1	3.0%	
3	109	56.8%	25	75.8%	
4	2	1.0%	1	3.0%	
4a	7	3.6%	1	3.0%	
4b	23	12.0%	5	15.2%	
**N**					0.770
0	116	60.4%	18	54.5%	
1	49	25.5%	9	27.3%	
2	27	14.1%	6	18.2%	
**M**					0.089
0	157	81.8%	23	69.7%	
1	35	18.2%	10	30.3%	
**Number of excised lymph nodes (median)**	19	14–24	18	12–28	0.724
**Adequate number of lymph nodes**					0.233
< 12	17	8.9%	5	15.2%	
≥ 12	125	91.1%	27	81.8%	
**Number of adjuvant chemotherapy cycles**	0	0–6	0	0–14	0.326

**Fig. 1:**
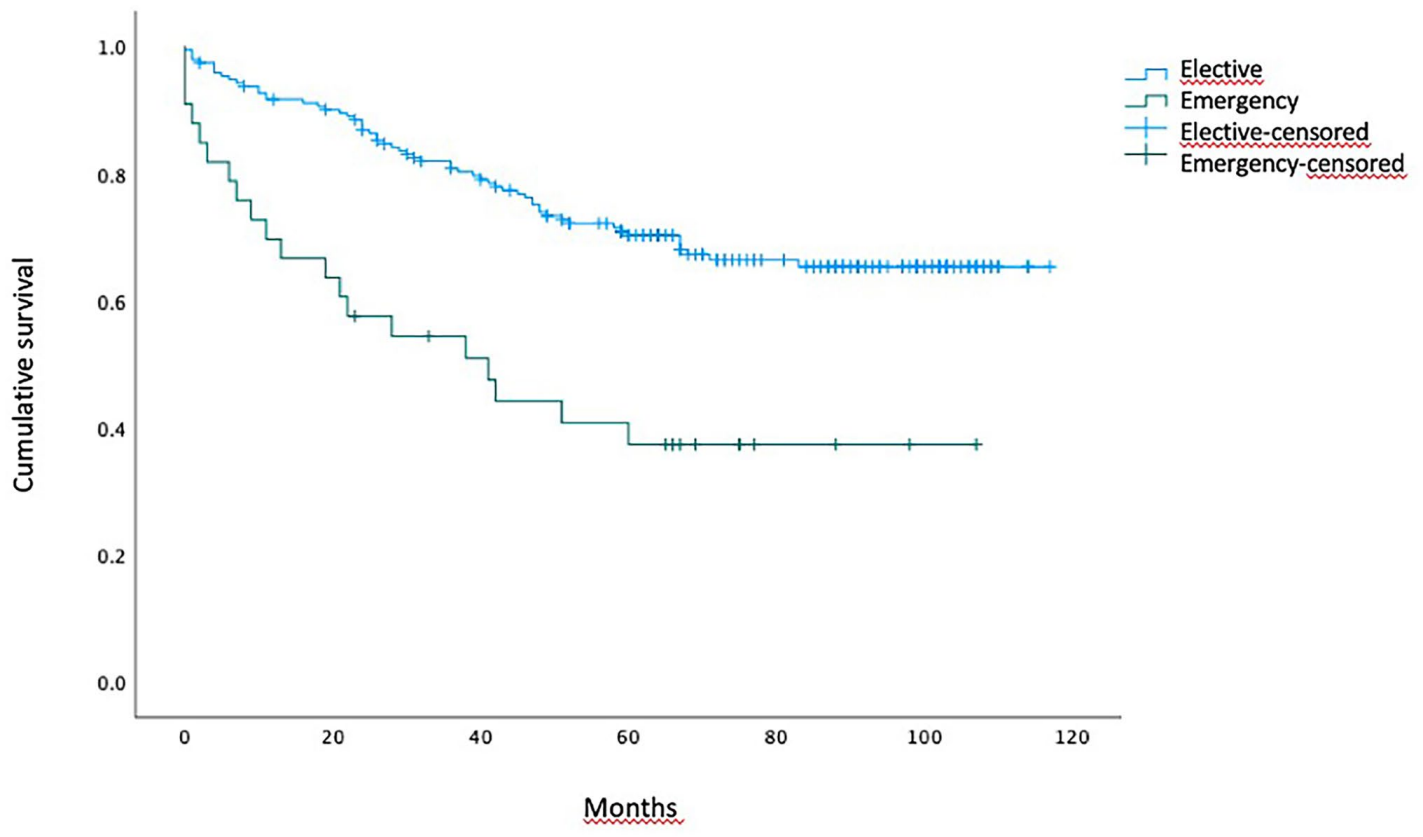
Overall survival. Median overall survival was 41.0 months 95%CI (15.1–66.9) in emergency resections vs. not reached in elective cases. Log-rank (Mantel-Cox) test, *p* < 0.001

At survival analysis, patients in the emergency group experienced significantly worse OS than the elective one (estimated median OS 41 months vs. not reached, for emergency and elective groups, respectively; *p* < 0.001 at Log-rank test) (Fig. 1).

**Fig. 2:**
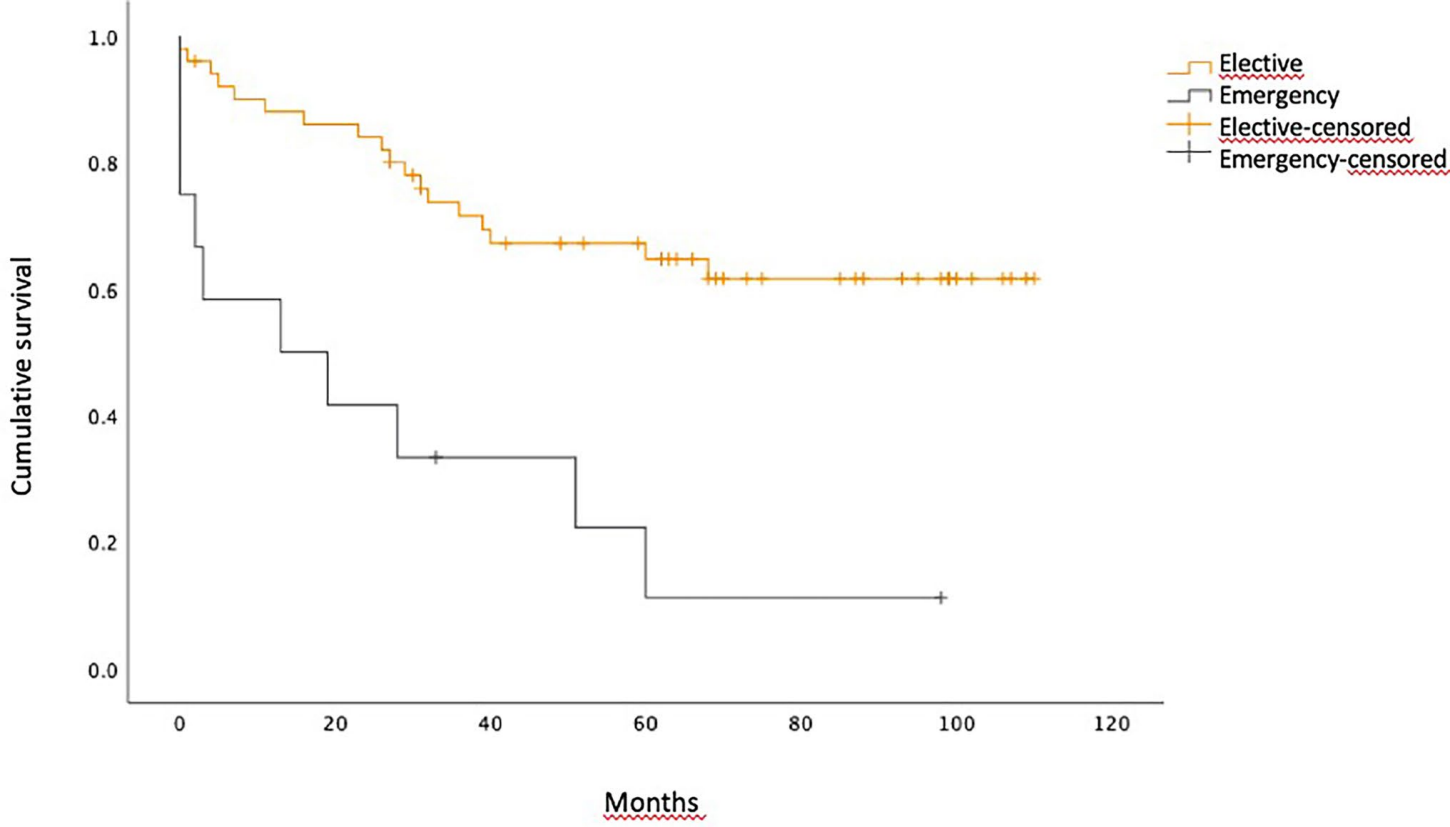
Overall survival in patients who experienced postoperative complications. Median overall survival was 13.0 months 95%CI (0.0–40.2) in emergency resections vs. not reached in elective cases. Log-rank (Mantel-Cox) test, *p* < 0.001

The same effect was observed at a subgroup analysis including patients who developed any complications, as shown in Fig. 2. The Kaplan-Meier analysis in patients with uneventful postoperative recovery showed much better OS, estimating more than 50% of patients alive at 120 months.

**Fig. 3:**
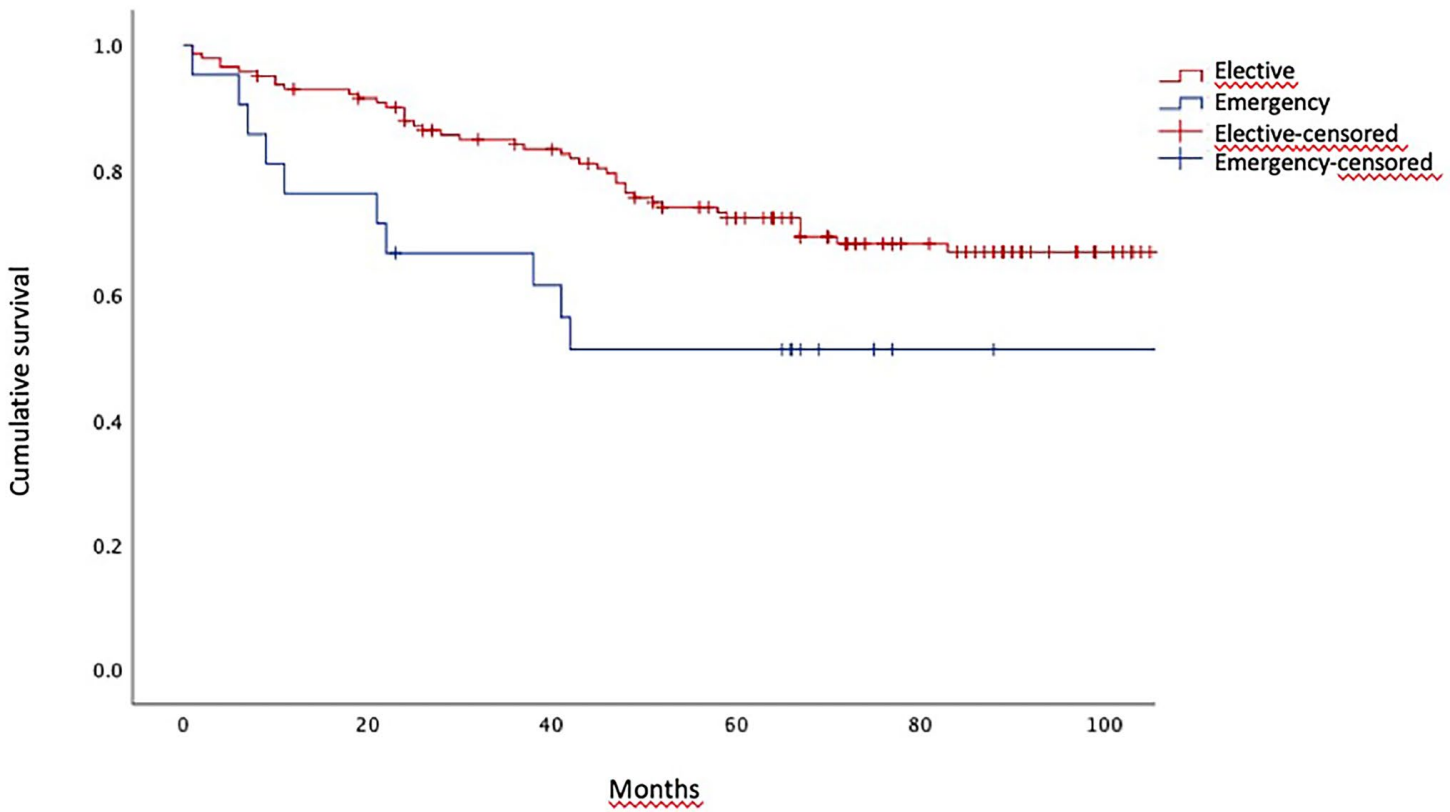
Overall survival in uncomplicated patients. In both group the estimated median overall survival was not reached. Log-rank (Mantel-Cox) test, p  = 0.035

However, the risk of death remained at each time point significantly higher in emergency operations vs. elective, with HR 2.07, 95%CI (1.04–4.13), as per Fig. 3.

At the multivariate Cox-proportional regression analysis, the emergency intervention remained independently associated with unfavorable long-term survival [HR 1.97; 95%CI (1.08–3.60), *p* = 0.028], together with the occurrence of major postoperative complications [HR 3.18; 95%CI (1.28–7.88); *p* = 0.012), the presence of distant metastasis [HR 5.85; 95%CI (2.70–12.70), *p* < 0.001), and the receipt of postoperative transfusions [HR 2.10; 95%CI (1.01–4.38), *p* = 0.049] (Table 4).

**Table 4:** Multivariate Cox-proportional regression analysis

	**HR**		**95% CI**		***p*-value**
			**Lower**	**Upper**	
**Elective/Emergency**	1.968		1.076	3.598	**0.028**
**Age**	1.048		1.019	1.078	**<0.001**
**Charlson Comorbidity Index**					0.164
3–4 vs. 0–2	1.455		0.163	12.982	0.737
5–7 vs. 0–2	1.732		0.209	14.331	0.611
≥ 8 vs. 0–2	3.023		0.341	26.801	0.320
**Major complications (Clavien Dindo ≥ 3)**	3.180		1.283	7.882	**0.012**
**Stage**					**< 0.001**
Stage 2 vs. 1	1.195		0.553	2.581	0.650
Stage 3 vs. 1	1.655		0.727	3.768	0.230
Stage 4 vs. 1	5.854		2.698	12.705	**< 0.001**
**Trasfusions**	2.096		1.003	4.380	**0.049**
**Permanent stoma**	0.891		0.335	2.369	0.817

## Discussion

The short-term surgical outcomes and overall survival (OS) in patients undergoing emergency versus elective surgery for colonic cancer remains controversial. Despite a bunch of evidence suggesting unfavorable effects of emergency operations, confounders such as reduced accuracy of the oncologic resection have been advocated as responsible for the worse long-term survival [16]. A recent systematic review and meta-analysis including a mixed population of colonic and rectal cancers showed a cumulative worse overall survival in patients who underwent emergency operations compared to elective cases, although reporting similar lymph-node harvesting [17]. Other mediators of dismal long-term survival have been also observed in the emergency setting, namely a higher occurrence of postoperative morbidity [18], more advanced AJCC stages [19], and severely impaired preoperative patient conditions [5].

In our retrospective analysis, we confirmed previous observations showing a reduced overall survival in patients undergoing emergency resection for colonic cancer. Specifically, patients receiving emergency colectomy had a significant worse median 41 months OS, vs. not reached in elective operations. The detrimental effect of emergency procedures observed in our series is similar to other findings [17].

Several factors may be potentially responsible for the observed unfavorable outcome. First, patients undergoing emergency surgery present with a systemic inflammatory response normally not detected in elective cases. In our series, the emergency was mainly conditioned by bowel occlusion, followed by perforation and bleeding. All these events are commonly associated with dehydration, electrolytes derangements, infection or sepsis, and impaired circulatory function. Therefore, we detected nearly double rate of ASA III-IV in the emergency group vs. elective. Moreover, patients in the emergency group were in median 8 years older and—despite a similar prevalence of comorbidities—computed overall higher Charlson Comorbidity Index scores. Second, we observed a significantly higher rate of major complications following surgery in the emergency group. The occurrence of postoperative major morbidity has been widely associated with reduced oncologic survival in many types of gastrointestinal malignancies [20–23]. Conversely, despite increased preoperative risk in the emergency group, no differences in term of overall morbidity, or medical morbidity have been reported. This is somehow in contrast with previous studies, suggesting generally higher rates of overall complications in emergency cases, but inconsistency if considering the occurrence of major morbidity [18]. However, other series considered more heterogeneous indications for surgery. Seeto and colleagues also included resections for diverticulitis. In the emergency group, they observed indeed a rate of complications 2-fold higher than in our emergency group, where only colonic cancers were included. Moreover, the mean age in the Australian cohort was about 10 years younger than our population. Even though the indication colectomy for infectious disease can explain an increased rate of overall morbidity, the younger age is generally associated with preserved resilience, and consequently with increased tolerance and better recovery from complications. We can thus partially justify the discordances in rates of overall and major postoperative complications compared to our series. However, as we observed a detrimental effect of emergency resection on long-term outcome also in uncomplicated patients, other underlying mechanisms responsible for the reduced long-term survival should be hypothesized.

Third, emergency operations are often associated with more advanced stages of the disease [19]. Bigger and spread tumors may reflect a more aggressive biological behavior and explain the unfavorable long-term prognosis. In our series, however, no significant differences in terms of final pathology have been described, and the multivariate analysis also confirmed that the elective surgical procedure affected long-term survival independent of the stage of disease.

It has also been advocated that the emergency setting may be responsible for reduced accuracy of the oncological standards, with reduced extent of lymphadenectomy and increased rate of R1 [19]. Despite the high heterogeneity among the studies, the meta-analysis by Zhou and colleagues showed similar number of harvested lymph-nodes in elective vs. emergency colorectal resections [17], according to our analysis. We also reported comparable rates of microscopic clearance of resection margins. From one side, these findings suggest that emergency colonic surgery is safe for the achievement of oncological standards in cancer patients. From the other side, however, no further etiologic explanation for the observed effect on survival may be retrieved.

In our study, the receipt of perioperative transfusion was significant and independent associated with dismal overall survival. It has been widely reported that the presence of anemia represents an unfavorable trait of colonic cancer patients [24–26], so that the preoperative screening and correction with iron administration represent nowadays the gold standard for the perioperative optimization of elective patients [27]. In our series, emergency patients did not receive the standard preoperative optimization and consequently the rate of perioperative transfusions was 3-fold higher. The detrimental effects of transfusions in resected colorectal cancer patients have been described [28–30]; however, this cannot represent the explaining mechanisms, as the emergency setting of the operations remained independently associated with survival at the multivariate analysis.

This study has some limitations. First, given the retrospective nature of the design, no sufficient information on the receipt of adjuvant chemotherapy were available for consideration as potential confounder. Moreover, our analysis included patients from a single center. Thus, despite the application of routinary procedures in accordance with international standards, our results should not be directly generalized. Finally, given the relatively small rate of severe postoperative complications, no subgroup Kaplan-Meier analysis was run according to the occurrence of major morbidity.

In conclusion, the emergency setting of colonic operation for cancer is associated with near double risk of death at any time-point following surgery. This effect has been observed independent of other well-known determinants of long-term survival, such as the occurrence of major postoperative morbidity, the AJCC stage of disease, and the receipt of perioperative transfusions. The actual mechanisms implied remains unclear and need further investigation. Until more evidence is provided, emergency colonic resection should be delayed in favor of elective surgery. Both the use of bridge stenting, when achievable, and the preoperative optimization of the patients should be a pursuit to improve postoperative short-term and oncologic outcomes.

## Data Availability

No datasets were generated or analysed during the current study.
